# Modulation-Specific and Laminar-Dependent Effects of Acetylcholine on Visual Responses in the Rat Primary Visual Cortex

**DOI:** 10.1371/journal.pone.0068430

**Published:** 2013-07-02

**Authors:** Shogo Soma, Satoshi Shimegi, Naofumi Suematsu, Hiroshi Tamura, Hiromichi Sato

**Affiliations:** 1 Graduate School of Frontier Biosciences, Osaka University, Toyonaka, Osaka, Japan; 2 Graduate School of Medicine, Osaka University, Toyonaka, Osaka, Japan; Medical University of South Carolina, United States of America

## Abstract

Acetylcholine (ACh) is secreted from cholinergic neurons in the basal forebrain to regions throughout the cerebral cortex, including the primary visual cortex (V1), and influences neuronal activities across all six layers via a form of diffuse extrasynaptic modulation termed volume transmission. To understand this effect in V1, we performed extracellular multi-point recordings of neuronal responses to drifting sinusoidal grating stimuli from the cortical layers of V1 in anesthetized rats and examined the modulatory effects of topically administered ACh. ACh facilitated or suppressed the visual responses of individual cells with a laminar bias: response suppression prevailed in *layers 2/3*, whereas response facilitation prevailed in *layer 5*. ACh effects on the stimulus contrast-response function showed that ACh changes the response gain upward or downward in facilitated or suppressed cells, respectively. Next, ACh effects on the signal-to-noise (S/N) ratio and the grating-phase information were tested. The grating-phase information was calculated as the F1/F0 ratio, which represents the amount of temporal response modulation at the fundamental frequency (F1) of a drifting grating relative to the mean evoked response (F0). In facilitated cells, ACh improved the S/N ratio, while in suppressed cells it enhanced the F1/F0 ratio without any concurrent reduction in the S/N ratio. These effects were predominantly observed in regular-spiking cells, but not in fast-spiking cells. Electrophysiological and histological findings suggest that ACh promotes the signaling of grating-phase information to higher-order areas by a suppressive effect on supragranular layers and enhances feedback signals with a high S/N ratio to subcortical areas by a facilitatory effect on infragranular layers. Thus, ACh distinctly and finely controls visual information processing in a manner that is specific for the modulation and cell type and is also laminar dependent.

## Introduction

Acetylcholine (ACh) plays an essential role in various brain functions including sensation [1], [2], attention [3]–[5], learning and memory [6]–[8], cognition [9], and regulation of the behavioral state [10]. A broad range of these modulatory effects is attributable to the characteristic features of the projection pattern of cholinergic neurons and the transmission mode of ACh [11]–[16]. For example, basal forebrain (BF) cholinergic neurons originating from the nucleus basalis project diffusely throughout the neocortex [11]–[13] and innervate nearly all cortical regions and layers [14]. Moreover, cholinergic fibers in the cortex have many axonal varicosities that do not associate with postsynaptic densities [15], suggesting ACh acts on neurons via a diffuse form of extrasynaptic modulation termed volume transmission [16]. Therefore, ACh has long been considered to exert a general and uniform modulatory effect across cortical areas and layers that regulates the higher-order brain functions required for cross-area communication.

The primary visual cortex (V1) is one target of cholinergic projections, and its visual information processing is modulated by ACh in many species including primate [17]–[21], tree shrew [22], cat [23]–[25], and rodent [26]. The modulatory effects of ACh have been mainly examined using local microionophoretic administration of ACh or agonists/antagonists of ACh receptors (AChRs). ACh facilitates or suppresses the visual responses of individual V1 neurons [17]–[26], and its modulatory effects are associated with changes in the gain of the contrast-response function [17], [18], [20], [22], [26]. Moreover, ACh has been known to change the selectivity of stimulus-features such as orientation, direction, and size [19], [21]–[25]. Thus, these ionophoretic studies demonstrated that ACh modulates various aspects of visual information processing by directly acting on local circuits in V1.

Goard and Dan [27] demonstrated that the activation of cholinergic neurons by electrical stimulation of the rat BF facilitates or suppresses visual responses in V1, with the response suppression prevailing in the supragranular layers. On the other hand, we recently found that microionophoretic administration of ACh in rat V1 causes no biased laminar distribution of the ACh effects [26]. One plausible explanation for the discrepancy is the ACh distribution. BF stimulation promotes wide release of ACh across all cortical layers in V1 as well as in other visual areas while microionophoretically administered ACh affects a limited local circuit within V1. Therefore, the laminar bias of the ACh effects by BF stimulation might be due to the direct effect of ACh on extensive networks within V1 or indirect effect on areas other of V1 via feedback inputs from those areas. To examine this point, we performed topical administration of ACh to rat V1 and measured the contrast-response relationship of the visual responses to a drifting grating stimulus by extracellular and simultaneous multi-point recordings across all layers.

Under ordinary vision, various visual images are randomly given to the retina to activate individual V1 neurons differentially in relationship with the receptive field properties and given stimulus-features, and ACh controls the gains of their neuronal activities for its optimization. To understand the functional role of ACh under the situation, a horizontal grating only was tested in this study, by which we examined how a single visual stimulus is processed by V1 neurons individually and collectively in different layers, and how ACh affects the information processing. The modulatory effects of ACh were assessed on 1) the response magnitude, 2) the laminar distribution, 3) the type of gain control, 4) the grating-phase information of the drifting grating, 5) the signal-to-noise (S/N) ratio, and 6) the type of cell (regular-spiking (RS) or fast-spiking (FS)).

We found that ACh facilitated or suppressed visual responses mainly in a manner of response gain control in individual cells. ACh-induced facilitatory modulation improved the S/N ratio of the visual responses, whereas the suppressive modulation strengthened the grating-phase dependency of the visual responses to drifting gratings. Interestingly, these ACh effects were observed in RS cells, but not FS cells. Moreover, ACh effects were distributed with a significant laminar bias where response suppression prevailed in *layers 2/3* and response facilitation prevailed in *layer 5*. Taking into account the fact that neurons in the supragranular and infragranular layers output their responses to cortical higher-order areas and subcortical nuclei, respectively, we concluded that ACh finely controls the visual information processing of V1 by sending output to functionally-differentiated areas in modulation-type- and cell-type-specific and laminar-dependent manners.

## Materials and Methods

All experimental protocols were approved by the Research Ethics Committee of Osaka University. All procedures were carried out in accordance with the regulations of the Animal Care Committee of the Osaka University Medical School and National Institutes of Health guidelines for the care of experimental animals (1996). All efforts were made to reduce the number of animals used.

### Preparation

Ten anesthetized male Long-Evans rats weighing 260–410 g were used to record neuronal responses in V1. Animal preparation procedures are described in detail elsewhere [28], [29]. Each animal was anesthetized by intraperitoneal (i.p.) injection of urethane (Kishida Chemical, Osaka, Japan; 1.5 g · kg^–1^) and supplemented as necessary. The animals were then placed in a stereotaxic apparatus, and their body temperature was maintained at about 37 °C by a heating pad. A local anesthetic, lidocaine, was administered at pressure points and around surgical incisions. The electrocardiogram, electroencephalogram, and heart rate were continuously monitored throughout the experiment.

### Simultaneous multi-point recordings and topical administration of ACh

We performed extracellular multi-point recordings and topical administration of ACh. The skull was exposed, and a small hole (less than 2 mm in diameter) was made above the monocular region of left V1 (coordinates: 1 mm anterior from the lambda and 3.5 mm lateral from the midline). A silicon polytrode (16 active channels separated by 150 µm; NeuroNexus Technologies, Ann Arbor, MI, USA) was inserted after removing a small portion of the dura. The wide-band signals were amplified, filtered, and collected by a computer running RASPUTIN (Plexon, Dallas, TX, USA) at 40 kHz.

For topical ACh administration, a microwell was made by gluing a plastic ring to the skull area surrounding the craniotomy. ACh (Nacalai Tesque, Kyoto, Japan; 1 mM, pH 7.0) was loaded into the microwell 5 min before and during the recording [1], [27]. After recording under ACh administration, ACh was washed out by Ringer's solution, and neuronal responses 5 min later were recorded in the Recovery condition (see Visual stimulation). At the end of each penetration, electrolytic lesions were produced by passing tip-negative direct current (intensity, 3–4 µA; duration, 10 s) from three separate channels. This enabled histological verification of the recording sites.

### Single-point recordings and microionophoretic administration of ACh

We performed extracellular single-unit recordings and microionophoretic administration of ACh to examine the relationship between ACh concentration and its modulatory effects. A glass microelectrode was attached to a two-barreled drug pipette in which the barrels were filled with ACh (Nacalai Tesque, Kyoto, Japan; 500 mM, pH 4.5) and Ringer's solution (pH 7.0) [20], [26]. The tip of the recording electrode protruded 10–30 µm from the tip of the drug pipettes. The ejecting current was generally between +1 and +100 nA, whereas the retaining current was between –5 and –15 nA. No cell showed any change in amplitude in spike waveforms or firing rate during the microionophoretic administration of Ringer's solution at the same range of ejecting currents. The recording pipette was filled with 0.5 M sodium acetate containing 4% Pontamine sky blue (Direct Blue 1; Tokyo Kasei, Tokyo, Japan). Dye marks were produced by passing tip-negative direct current at the end of each penetration (100–200 pulses of 8–10 µA at 0.5 Hz) for histological verification of the recording sites.

### Visual stimulation

A full-screen stimulus of drifting sinusoidal grating was generated by custom-made MATLAB (Mathworks, Natick, MA, USA) programs with Psychtoolbox [30], [31] and presented for 1 s monocularly on a CRT display monitor (CDM-F520; Sony, Tokyo, Japan; mean luminance, 30 cd/m^2^; refresh rate, 100 Hz; screen size, 40 × 30 cm^2^) placed 24 cm in front of the right eye. The right eye was fixed with a metal ring to prevent eye movement and irrigated with sterile saline [27], [32].

A grating stimulus with horizontal orientation at varying stimulus contrasts was used to obtain a contrast-response function. The spatial and temporal frequencies were 0.02–0.2 cycles/degree and 1–2 Hz, respectively. The neuronal response was measured while pseudorandomly changing the stimulus contrast, which included 9 contrast levels spanning 0–100%. Background discharge was defined as the spike discharge during the presentation of a blank stimulus with 0% contrast. Each stimulus presentation was interleaved with a blank screen with 0% contrast for 1 s. Each stimulus condition was pesudorandomly repeated 10 times to construct a peri-stimulus time histogram (PSTH). Measurements were performed before, during, and after ACh administration, which are referred to as the Control, ACh, and Recovery conditions, respectively.

### Histology

After the recording experiments, animals were deeply anesthetized with sodium pentobarbital (Nembutal; Dainippon Sumitomo Pharma, Osaka, Japan; 200 mg · ml^–1^ · kg^–1^, i.p.) and perfused transcardially with 0.1 M phosphate-buffered saline (PBS; pH 7.4) followed by 4% paraformaldehyde in 0.1 M PBS. Whole brains were obtained and immersed in 30% sucrose in PBS for 36–48 h. Sixty-micrometer-thick frozen parasagittal sections were sliced on a microtome and kept in PBS. Sections were stained for cytochrome oxidase [33]. The laminar positions of the recording sites were then identified under a light microscope. Shrinking in the cortical tissues was corrected by taking the ratio of the measured distance of electrolytic lesions and the distance between the channels used for making the electrolytic lesions for multi-point recordings or by taking the ratio of the measured dye mark distance and the distance calculated from the micrometer reading for single-point recordings [20], [26].

### Off-line spike sorting

Raw signal data (wide-band signals) were preprocessed using a custom-developed program with MATLAB. First, signals were down-sampled to 20 kHz and then band-pass filtered (0.5–5 kHz). Next, the spike signals with power >5 times the standard deviation from the baseline mean were extracted. To isolate single-unit activity from multi-unit activity, spike signals were processed by principal component analysis to extract the features of the spike waveforms and then automatically sorted by KlustaKwik, an automatic spike-sorting program [34]. Next, clusters of the sorted spikes were combined, divided or discarded manually to refine single-neuron clusters by Klusters, a powerful and easy-to-use cluster cutting application [35].

### Cell Classification

Two types of cells, RS and FS, were classified on the basis of the following spike waveform properties: amplitude (the ratio of trough-to-peak amplitude), time-course of spikes (trough-to-peak time), and end slope (slope at 0.25 ms after the trough of the waveform) [36]. The two populations were clearly separated and classified as RS (*n* = 108) or FS (*n* = 45) cells. RS and FS cells showed significantly different values for amplitude (mean ± SEM; RS: 0.31 ± 0.02; FS: 0.62 ± 0.03; *P*<0.001, unpaired *t*-test), time-course (mean ± SEM; RS: 0.40 ± 0.002 ms; FS: 0.27 ± 0.003 ms; *P*<0.001, unpaired *t*-test), and end slope (mean ± SEM; RS: 0.04 ± 0.07; FS: – 0.33 ± 0.04; *P*<0.001, unpaired *t*-test).

In this study, simple and complex cells were not classified according to the classical method based on the ratio between the amplitude of the first harmonic of the response and the mean spike rate [37], because the receptive fields were not stimulated with a grating of optimal parameters for individual neurons. Owing to rodent V1 having no orientation columns [38], the horizontal grating stimulus used in the present work would have activated neighboring cells or cells in different layers at various degrees in the relationship with their orientation preference.

### Assessments of ACh effects on visual responses

To examine how ACh modulates visual information processing, we assessed the effects on the: 1) response magnitude (response modulation), 2) contrast-response relationship (gain control), 3) phase information of drifting sinusoidal grating, and 4) S/N ratio.

#### Classification of ACh-induced response modulation

Response magnitude was defined as the number of spikes evoked during a stimulus presentation. The effects of ACh on the response magnitude were categorized to response facilitation and suppression according to a nonparametric analysis method described previously [17], [18], [20], [22], [26]. Briefly, we calculated and compared the areas under the contrast-response curves (response area) by summing the visual responses obtained from nine different contrast stimuli for the Control and ACh conditions. The statistical significance of the differences was determined by the Mann-Whitney U-test (α = 0.05), and significant increase and decrease in the response area by ACh was classified as facilitation and suppression, respectively. This analysis allowed us to evaluate the response modulation type induced by ACh independently of the three gain control types described in the following section.

#### Fitting of the contrast-response curve and three types of gain control

To quantify the contrast sensitivity of the recorded neurons, we fitted the contrast-response relationship using the following equation (Naka-Rushton function [39]): R = R_max_ · C^n^/(C^n^+C_50_^n^)+b, where R is the neuronal response, C is the contrast of grating stimuli, and b is the background discharge. R_max_ (maximal response), n (exponent of the power function, >0) and C_50_ (contrast at half R_max_) are free parameters.

**Figure 1:**
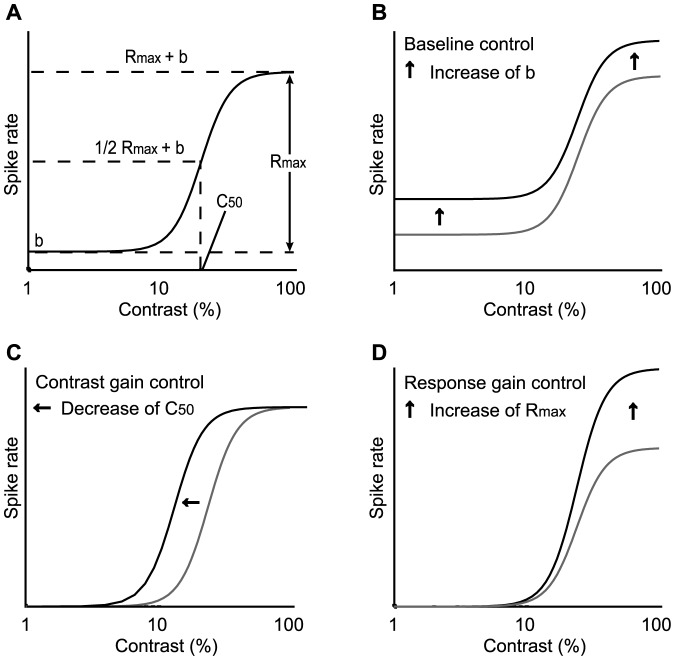
Naka-Rushton function and the three possible types of gain control in the contrast-response function. A: The contrast-response tuning curve of each neuron was fitted using the Naka-Rushton function, where R_max_ is the peak (maximal) response, b the background discharge measured during the presentation of a blank stimulus with 0% contrast, and C_50_ the contrast value at 1/2 R_max_ (contrast sensitivity). B–D: Three kinds of gain control are considered: baseline control (B), contrast gain control (C), and response gain control (D), which are characterized by changes in b, C_50_, and R_max_, respectively, as depicted by the arrows.

Figure 1A is an explanatory schema of a contrast-response curve represented as the Naka-Rushton function. There are at least three possible types of gain control in the contrast-response function: baseline control (Fig. 1B), contrast gain control (Fig. 1C), and response gain control (Fig. 1D) [40], [41]. They are discriminable by a change in the parameters of the Naka-Rushton function. Baseline control is a contrast-independent modulation characterized by a change in b. On the other hand, both contrast gain control and response gain control are contrast-dependent modulations marked by changes in C_50_ and R_max_, respectively. To examine which type of gain control occurred in the modulated cells, we compared the fitting parameters obtained from the Control and ACh conditions.

#### Phase information of drifting grating

Information about the phase of drifting sinusoidal grating is reflected in the temporal structure of the visual responses consisting of a phase-dependent modulated response and a phase-independent unmodulated response. To examine whether and how ACh influences the encoding of the grating-phase information, we evaluated the modulated and unmodulated responses as the amount of temporal response modulation at the fundamental frequency (F1) of a drifting grating and the mean evoked response (F0), respectively, from PSTHs obtained by 10 repetitions of stimulation using the discrete Fourier transform. The F1/F0 ratio was calculated as a measure of the neuronal representation of the grating-phase information.

#### S/N ratio

To assess whether ACh facilitates distinguishing the presence or absence of a signal, the S/N ratio was calculated as follows [21], [24]: S/N ratio = R_stim_/(R_stim_+R_spont_), where R_stim_ is the stimulus-driven response, and R_spont_ is the spontaneous activity that is recorded during the pre-stimulus period (1 s).

## Results

To understand how ACh widely diffusing in V1 modulates cortical visual information processing, we performed multi-point extracellular recordings of neuronal activities across all cortical layers of V1 before, during, and after the topical administration of ACh in anesthetized rats. Among the 227 neurons identified with off-line spike sorting, 153 showed significant visual responses to the grating stimuli (*P*<0.05, Mann-Whitney U-test).

### Cholinergic response modulation

**Figure 2:**
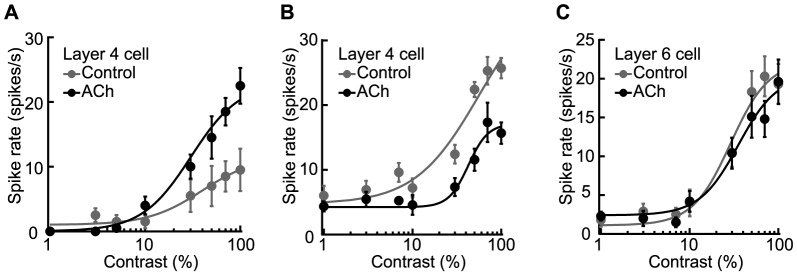
Effects of ACh on the visual responses of neurons in V1. A–C: Fits were obtained for contrast-response functions under no drug condition (Control: gray dots and line) and ACh administration (ACh: black dots and line). Visual responses were facilitated (A), suppressed (B), and not affected (C). Modulatory effects were quantitatively classified by comparing the areas under the contrast-response functions (response area; see Materials and Methods). Error bars = SEM.

We first examined ACh effects on the magnitude of the visual responses (response modulation). Topical administration of ACh increased (Fig. 2A), decreased (Fig. 2B), and had no effect (Fig. 2C) on the visual responses of individual neurons. Quantitative classification of the ACh effects was conducted by comparing the response areas, which were defined as the areas under the contrast-response curves, between the Control and ACh conditions (see Materials and Methods). Among the 153 recorded cells, 53 (35%), 49 (32%), and 51 (33%) were categorized as facilitated, suppressed, and no-effect cells, respectively. The different ACh effects may be due to the type of cell, such as excitatory or inhibitory. Therefore, we examined the relationship between the response modulation type and RS and FS cells, where RS (*n* = 108) and FS (*n* = 45) cells were classified electrophysiologically based on their spike waveforms (see Materials and Methods). Among the RS cells, 43 (40%), 32 (30%), and 33 (30%) were facilitated, suppressed, and no-effect cells, respectively, while among the FS cells the numbers were 10 (22%), 17 (38%), and 18 (40%), respectively. Thus, there were no statistical differences in the occurrence rate of ACh effects between the two cell types (*P* = 0.114, χ^2^ test), suggesting that the type of response modulation is independent of the type of cell, excitatory or inhibitory.

### Laminar distribution of ACh effects in V1

**Figure 3:**
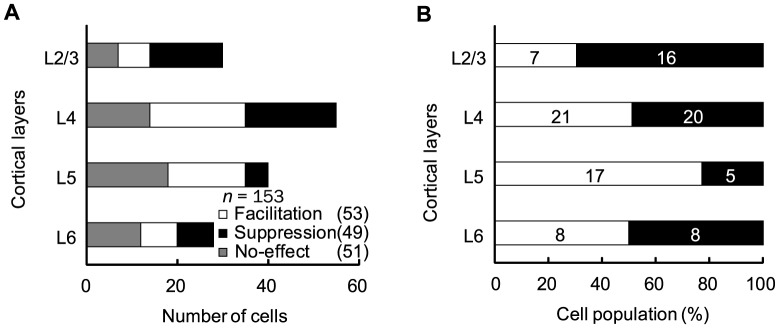
Laminar (L) distribution of ACh effects in V1. A: V1 neurons (*n* = 153) were reconstructed from the laminar positions of the recording sites on the basis of histological observations. Each column shows the number of cells facilitated (open columns), suppressed (filled columns), and unaffected (gray columns) by topical ACh administration. B: Percentage of modulated neurons for each layer (*n* = 102). Open and filled columns indicate the percentages of facilitated and suppressed cells, respectively (the number of observed cells is shown in each column). The laminar distributions of ACh effects were significantly different (*P*<0.05, χ^2^ test), with response suppression and facilitation being predominantly observed in *layers 2/3* and *layer 5*, respectively, while no bias was found in the other layers.

Goard and Dan [27] found the laminar bias of the ACh effects in rat V1 under electrical stimulation of BF, while our recent study observed no bias when ACh was administered microionophoretically [26]. The discrepancy between the two studies seems to be due to the difference of ACh distribution. If the laminar bias of the ACh effect results from the direct action on extensive networks within V1, ACh widely diffused into V1 through topical administration should cause the similar laminar bias. To examine this point, we histologically reconstructed the laminar positions of the recording sites for all cells (*n* = 153). Interestingly, the response facilitation and suppression were not equally distributed throughout the cortical layers (*P*<0.05, χ^2^ test; Fig. 3). The percentage of response suppression was high in *layers 2/3* (70%, 16/23 cells), whereas response facilitation was predominantly observed in *layer 5* (77%, 17/22 cells). Thus, the laminar bias of the ACh effects induced by topical administration was consistent with those observed by electrical stimulation of BF [27].

### Validity of topical administration

As shown in Figures 2 and 3, topical administration of ACh caused both response facilitation and suppression, but with a laminar bias. To confirm these results were not due to the uneven distribution of ACh administered topically, we performed the following two experiments.

**Figure 4:**
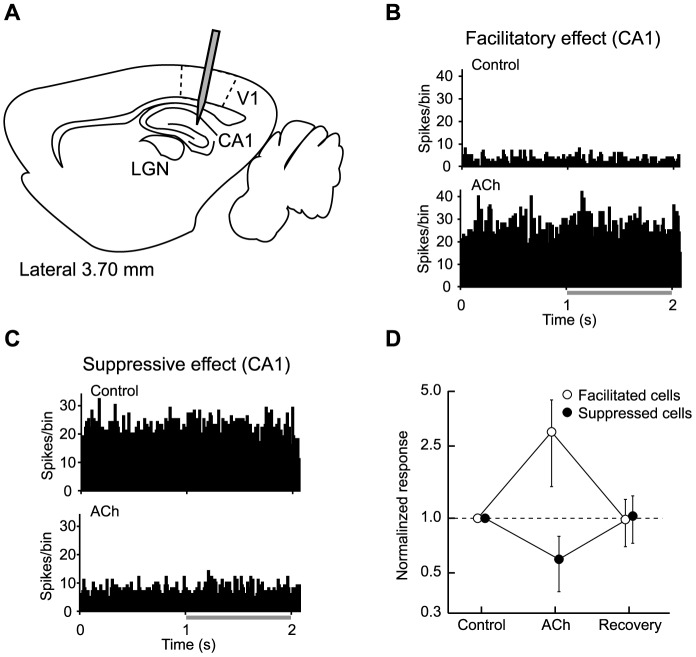
ACh effects on hippocampal neurons confirm its diffusion into V1 deep layers. A: Schematic illustration of topical ACh administration and multi-point recordings. A silicon polytrode was inserted into all cortical layers of V1 in order to simultaneously record neuronal activities from different layers. The tip of the electrode was positioned at CA1 in the hippocampus to check whether ACh administered from the surface of V1 sufficiently diffused into the deep layer of V1. B–C: PSTHs (trials, 10; bin width, 10 ms) of neuronal activities recorded from CA1 neurons. Top and bottom indicate the PSTHs obtained under the Control and ACh conditions, respectively. The gray underline indicates the visual stimulation period (1 s). Topical administration of ACh increased (B) or decreased (C) the number of spikes irrespective of the visual stimulus, suggesting that topically administered ACh reached the CA1 through V1 and white matter. D: ACh effects on the neuronal activities of CA1 neurons. Open and filled circles show ACh-induced facilitated (*n* = 7) and suppressed (*n* = 6) neurons, respectively. ACh-induced changes in activities returned to pre-ACh levels after ACh washout (facilitated cells, *P*<0.01; suppressed cells, *P*<0.05, Wilcoxon signed-rank test). V1, primary visual cortex. CA1, cornu ammonis area 1. LGN, lateral geniculate nucleus. Error bars = SEM.

First, we checked whether ACh administered from the surface of V1 sufficiently diffused into the deep layer of V1 by simultaneously recording neuronal activities from both V1 and cornu ammonis area 1 (CA1) (Fig. 4A). Figure 4B and 4C show the PSTHs of the neuronal activities from two CA1 neurons. Neither neuron responded to the presence of a visual stimulus, but both were sensitive to the topical administration of ACh, where neuronal activities were facilitated (Fig. 4B) or suppressed (Fig. 4C). In either case, the neuronal activities recovered to their original level after ACh washout (facilitated cells, *n* = 7, *P*<0.01; suppressed cells, *n* = 6, *P*<0.05, Wilcoxon signed-rank test; Fig. 4D). Thus, this experiment confirmed that topically administered ACh reached the deep cortical layers.

**Figure 5:**
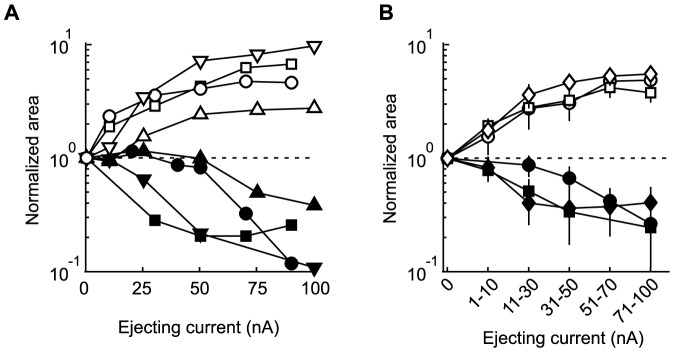
The direction of ACh effects was independent of the ejecting current. We performed extracellular single-unit recordings with microionophoretic ACh administration to examine the relationship between ACh concentration and the modulatory effects. Varying ejecting current levels were tested, and the ejecting current-response curve was obtained. Areas under the contrast-response curves (response area) were normalized to the response area of the Control condition and were plotted against the ejecting current. Open and filled symbols show facilitated and suppressed neurons, respectively. A: Typical examples. Neurons were recorded across all cortical layers: *layers 2/3* (circles), *layer 4* (squares), *layer 5* (triangles), and *layer 6* (inverted triangles). B: Population average. Circles, squares, and diamonds represent neurons recorded from supragranular, granular, and infragranular layers, respectively. Increasing the ejecting current equivalent to ACh concentration strengthened the ACh-induced facilitatory and suppressive effects, but did not change the direction of the response modulation, suggesting that ACh affects the visual responses of each cell uni-directionally. Error bars = SEM.

Next, we performed single-unit recordings and local administration of ACh by microionophoresis in order to investigate the relationship between the concentration of ACh and the modulatory effects (see Materials and Methods). We tested varying ejecting current levels and obtained the ejecting current-response curve (Fig. 5). The response areas were normalized by those of the Control condition and plotted against the ejecting current. Figure 5A shows typical examples of four facilitated (opened symbols) and four suppressed (filled symbols) neurons that were recorded from *layers 2/3* (circles), *layer 4* (squares), *layer 5* (triangles), and *layer 6* (inverted triangles). Increasing the ejecting current (the ACh concentration) strengthened the ACh-induced facilitatory and suppressive effects, but did not change the direction of the response modulation across all cortical layers. Figure 5B shows the population average in each layer (supragranular layers, circles, facilitation, *n* = 6, suppression, *n* = 5; granular layer, squares, facilitation, *n* = 6, suppression, *n* = 5; infragranular layers, diamonds, facilitation, *n* = 6, suppression, *n* = 6). Consistent with the typical examples in Fig. 5A, ACh caused concentration-dependent monotonic effects on the response area, suggesting that ACh affects visual responses uni-directionally in individual cells, and the type of response modulation is not linked to the ACh concentration.

### Cholinergic gain control of visual responses

**Figure 6:**
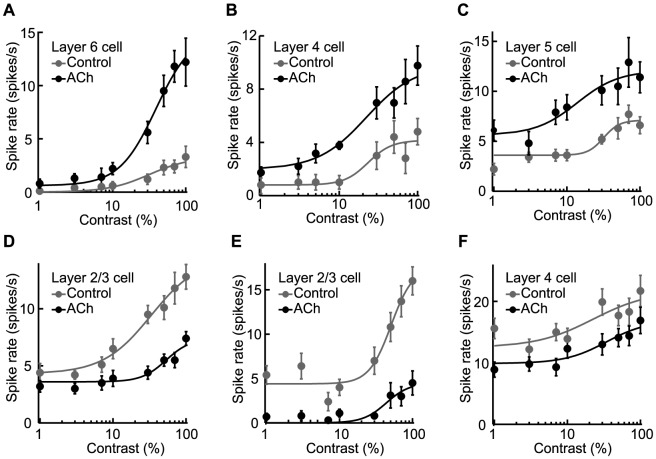
Contrast-dependent and -independent gain controls by ACh. A–F: Fits were obtained for contrast-response functions under the no drug condition (Control: gray dots and line) and ACh administration (ACh: black dots and line). Visual responses were contrast-dependently facilitated (A) or suppressed (D) and showed response gain control. On the other hand, ACh changed the background discharge, resulting in upward (C) or downward (F) shifts, which indicate contrast-independent gain control, i.e. baseline control. Some neurons were modulated by ACh in both a contrast-dependent and -independent manner (B and E). Error bars = SEM.

ACh has long been suggested to be responsible for the gain control of a visual response. To examine this effect, we examined ACh-induced changes in the shape of the contrast-response curves, finding a variety of changes in individual cells (Fig. 6). Figure 6A and 6D are examples of ACh-induced response gain control, where the visual responses were enhanced (Fig. 6A) or suppressed (Fig. 6D) in proportion to the magnitude of the control response, which is categorized as a contrast-dependent modulation. On the other hand, Figure 6C and 6F are examples of baseline control, showing that ACh shifted the contrast-response curve upward (Fig. 6C) or downward (Fig. 6F) over the whole range of the stimulus contrasts independent of the stimulus contrast. Thus, the response gain control and baseline control are distinguished as contrast-dependent and -independent gain controls. However, the modulatory effects cannot be simply classified according to the contrast dependency of the gain control, since there were neurons that showed both response gain control and baseline control (Fig. 6B and 6E). The cells represented in Fig. 6B and 6E were facilitated or suppressed contrast-dependently in addition to an increase or decrease in spontaneous discharges, respectively.

**Figure 7:**
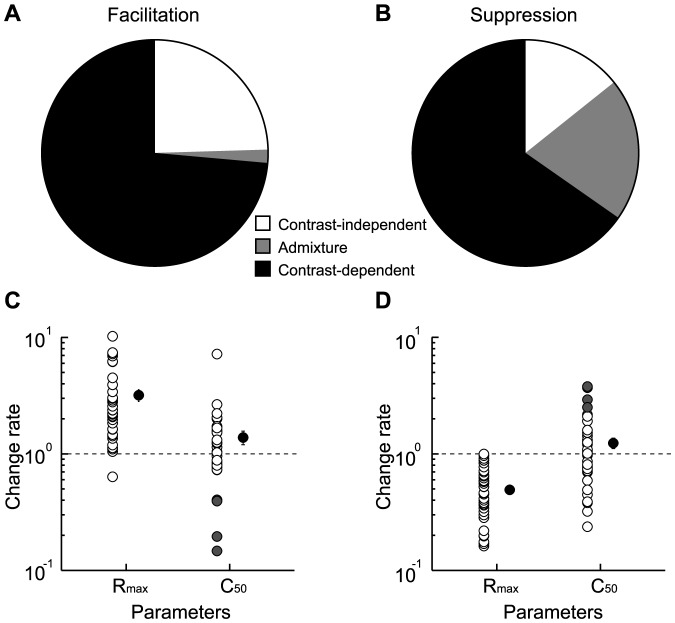
Response gain control by ACh. A–B: Pie chart illustrating the proportion of ACh modulatory effects on gain control in facilitated (A) and suppressed (B) cells. V1 neurons were predominantly facilitated (75%) or suppressed (86%) in a contrast-dependent manner (filled + gray), but some neurons were contrast-independently modulated, meaning that ACh changed the background discharge only (open; facilitated cells: 25%; suppressed cells: 14%). C–D: Population data of R_max_ and C_50_ obtained from cells showing response facilitation (C, *n* = 40) or suppression (D, *n* = 42) by ACh except for those that showed baseline control only. Gray circles indicate facilitated and suppressed cells whose C_50_ were decreased or increased by more than 1 SD from the population average, respectively (C, facilitated cells, *n* = 4; D, suppressed cells, *n* = 7). Error bars = SEM.

To determine what percentage of modulated cells (*n* = 102) showed contrast-independent modulation, we compared ACh effects on background discharge (Fig. 7A and 7B). A significant increase or decrease in background discharge was observed in 14 of the 53 facilitated cells and 17 of the 49 suppressed cells (*Ps*<0.05, Mann-Whitney U-test). Thus, baseline control was shown in 31 ( = 14+17) modulated cells, and the remaining 71 cells showed contrast-dependent gain control only (filled; facilitated cells, *n* = 39; Fig. 7A; suppressed cells, *n* = 32; Fig. 7B). The 31 cells showing baseline control may also have contrast-dependent gain control, like the cells depicted in Figure 6B and 6E. To investigate whether the response modulations were attributable to baseline control only, we subtracted the background discharge from the visual response and compared the results between Control and ACh conditions. If ACh caused baseline control only, its facilitatory/suppressive effects should disappear after the subtraction. This indeed occurred in 13 of the 14 facilitated and 7 of the 17 suppressed cells (open; Fig. 7A and 7B), indicating that the remaining 11 cells (gray; facilitated cells, *n* = 1; Fig. 7A; suppressed cells, *n* = 10; Fig. 7B) had contrast-dependent gain control coincident with baseline control (see Fig. 6B and 6E). Therefore, the contrast-dependent gain control was observed in 80% (82 = 11+71) of all cells (*n* = 102) modulated by ACh, while the remaining 20% (20/102) had contrast-independent modulation only.

Contrast-dependent gain control is classified as contrast gain control (Fig. 1C) or response gain control (Fig. 1D). To examine which gain control contributes more dominantly in our samples (*n* = 82), we compared the changes in R_max_ and C_50_ between Control and ACh conditions. Figure 7C and 7D represent ACh-induced changes in the R_max_ and C_50_ values of facilitated (*n* = 40; Fig. 7C) and suppressed (*n* = 42; Fig. 7D) cells. ACh significantly increased and decreased R_max_ (*Ps*<0.001, Mann-Whitney U-test) by factors of 3.1 and 0.5 in facilitated and suppressed cells, respectively (mean ± SEM; change rate, facilitated cells: 3.1 ± 0.4; suppressed cells: 0.5 ± 0.1). On the other hand, C_50_ showed a large variation, increasing or decreasing in either facilitated or suppressed cells (mean ± SEM; change rate, facilitated cells: 1.4 ± 0.2; suppressed cells: 1.2 ± 0.1). Neither the facilitatory nor suppressive effects on C_50_ were statistically significant (facilitated cells, *P* = 0.462; suppressed cells, *P* = 0.421, Mann-Whitney U-test). Thus, the ACh-induced contrast-dependent effects are mostly attributable to response gain control.

In a certain population of cells, ACh-induced changes in C_50_ seemed to contribute to contrast-dependent facilitation or suppression, as C_50_ decreased (facilitated cells, gray circles, *n* = 4; Fig. 7C) or increased (suppressed cells, gray circles, *n* = 7; Fig. 7D) by a value larger than 1 SD from the population average. These cells were distributed across all cortical layers (supragranular layers, *n* = 4, granular layer, *n* = 4, infragranular layers, *n* = 3) and were observed in both RS (*n* = 6) and FS (*n* = 5) types. In all cells that showed contrast-dependent modulation (*n* = 82), the statistical significance of the rate of change in C_50_ was assessed, and neither RS (*n* = 60) nor FS (*n* = 22) cells showed a significant change (mean ± SEM; change rate, RS: 1.5 ± 0.2, *P* = 0.114; FS: 1.2 ± 0.1, *P* = 0.252, Mann-Whitney U-test). We also examined the relationship between the change rate and spontaneous activity, but found no significant relationship (*R* = –0.06, Pearson's correlation coefficient; *P* = 0.546, *t*-test of the correlation coefficient). Thus, the variability of the ACh-induced change in C_50_ was not related to the laminar location, cell type, or intrinsic firing properties.

### Cholinergic effects on the cortical processing of visual signals

To understand the functional roles of ACh from the viewpoint of signal processing, we analyzed the effects of ACh on grating-phase information processing and the S/N ratio. Using discrete Fourier transforms, the neuronal representation of the phase information of drifting grating was evaluated as the F1/F0 ratio, i.e., the content ratio of the phase-dependent response (F1) to the total response (F0). The S/N ratio was assessed as the neuronal ability to detect signals embedded in noise such as spontaneous discharge.

**Figure 8:**
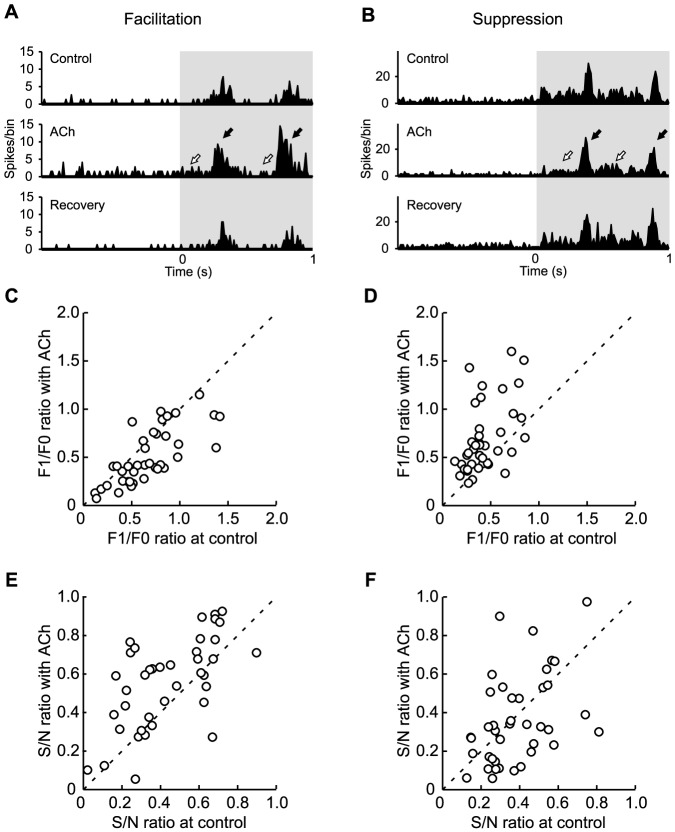
Cholinergic effects on the F1/F0 and S/N ratios in facilitated and suppressed cells. A–B: Each histogram shows a PSTH (trials, 10; bin width, 10 ms) of visual responses of two neurons to a drifting sinusoidal grating presented for 1 s (gray area). Examples of neurons facilitated (A) and suppressed (B) by ACh. Top, middle, and bottom show visual responses obtained before (Control), during (ACh), and after (Recovery) ACh administration, respectively. C–F: Population data of the F1/F0 and S/N ratios. All cells showing contrast-dependent gain modulation by ACh administration were analyzed (C and E, facilitated cells, *n* = 40; D and F, suppressed cells, *n* = 42).

Figure 8A and 8B illustrate typical examples of temporal response patterns in a single facilitated (Fig. 8A) or suppressed cell (Fig. 8B) under Control (top), ACh (middle), and Recovery (bottom) conditions. PSTHs were constructed for three stimulus contrasts (50%, 70%, and 100%) that each evoked sufficient visual responses for the analysis of the above parameters. In these examples, a drifting grating was presented at a temporal frequency of 2 Hz, which caused the cell to respond vigorously in a manner that corresponded to the spatial phase of grating under the Control condition. The F1/F0 and S/N ratios were differentially affected by ACh in both facilitated and suppressed cells. In the facilitated cell, ACh administration increased the total number of spikes during visual stimulation to 206% that of the Control condition (Fig. 8A), with the modulated responses (black arrows) and unmodulated responses (white arrows) being enhanced by ACh. Additionally, the F1/F0 ratio decreased to 0.8 times, and the S/N ratio increased 2.2 times relative to Control. These effects suggest that ACh regulates cells to predominantly output information about the presence of a stimulus rather than information about the phase of grating by facilitatory response modulation. On the other hand, in the suppressed cell (Fig. 8B), ACh administration decreased the total number of spikes during visual stimulation to 66% that of the Control response, where the unmodulated responses (white arrows) were more strongly suppressed than the modulated responses (black arrows). This caused a 2.5 fold increase in the F1/F0 ratio, suggesting that the suppressive modulation of ACh enhances the strength of the grating-phase signal. Since ACh also suppressed spontaneous discharges, the S/N ratio was not markedly changed (1.1 times).

The population data also shows modulation-type-specific ACh effects on the F1/F0 (Fig. 8C and 8D) and S/N ratios (Fig. 8E and 8F). The cells showing baseline control only were excluded from this analysis, because their visual responses were not modulated by ACh. Figure 8C and 8E represent the results of facilitated cells with contrast-dependent modulation (*n* = 40). ACh significantly decreased the F1/F0 ratio, but increased the S/N ratio (F1/F0 ratio, *P*<0.05; S/N ratio, *P*<0.05, Mann-Whitney U-test). On the other hand, in suppressed cells with contrast-dependent modulation (*n* = 42), ACh significantly increased the F1/F0 ratio (*P*<0.001, Mann-Whitney U-test; Fig. 8D), suggesting that unmodulated responses are more dominantly suppressed than modulated responses. The effect of ACh on the S/N ratio varied widely from cell to cell and was not statistically significant (*P* = 0.434, Mann-Whitney U-test; Fig. 8F). Thus, suppressive response modulation seems to act more as a signal amplifier than gain controller.

**Figure 9:**
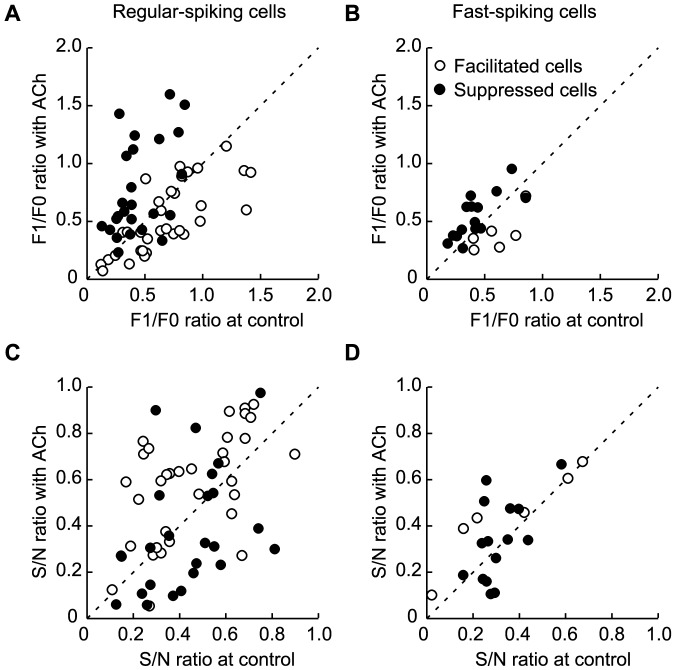
Cholinergic effects on the F1/F0 and S/N ratios in RS and FS cells. A–D: Population data of the F1/F0 (A, B) and S/N ratios (C, D). All cells showing contrast-dependent gain control by ACh administration were analyzed (A and C, RS, *n* = 60; B and D, FS, *n* = 22). Facilitated and suppressed cells are indicated by open and filled circles, respectively.

Finally, we conducted the same analysis for each cell type, RS (*n* = 60; Fig. 9A and 9C) and FS (*n* = 22; Fig. 9B and 9D), and for each modulation type (facilitated cells, open circles; suppressed cells, filled circles). Statistical analyses of ACh effects were performed for four groups: 1) facilitated RS cells (*n* = 34), 2) suppressed RS cells (*n* = 26), 3) facilitated FS cells (*n* = 6), and 4) suppressed FS cells (*n* = 16). Interestingly, in the suppressed cells (filled circles), ACh increased the F1/F0 ratio in the RS (Fig. 9A), but not the FS (Fig. 9B) cells (suppressed RS, *P*<0.001; suppressed FS, *P* = 0.110, Mann-Whitney U-test). Similarly, in the facilitated cells (open circles), ACh improved the S/N ratio in the RS (Fig. 9C), but not the FS (Fig. 9D) cells (facilitated RS, *P*<0.05; facilitated FS, *P* = 0.589, Mann-Whitney U-test). Thus, the F1/F0 and S/N ratios of visual responses were significantly modulated in RS cells, which are presumed to be output cells in V1, but not in FS cells, which are presumed to be inhibitory interneurons.

When the statistical analysis of ACh effects were performed for each cell type without consideration of the modulation type, neither the F1/F0 ratio (RS, *P* = 0.518; FS, *P* = 0.859, Mann-Whitney U-test; Fig. 9A and 9B) nor S/N ratio (RS, *P* = 0.390; FS, *P* = 0.09, Mann-Whitney U-test; Fig. 9C and 9D) showed significant change.

## Discussion

The main results of the present study are as follows: 1) ACh facilitated or suppressed the neuronal activity in V1 of rats, 2) ACh effects were observed across all cortical layers with a laminar bias in which the response suppression and facilitation was predominantly observed in *layers 2/3* and *layer 5*, respectively, 3) ACh changed the gain of the contrast-response relationship mainly in a manner of response gain control, 4) the facilitatory response modulation improved the S/N ratio, whereas the suppressive response modulation enhanced the F1/F0 ratio without a significant reduction in the S/N ratio, and 5) ACh effects on the F1/F0 and S/N ratios were observed in RS cells, but not in FS cells.

### Modulation type and its laminar bias

We demonstrated that topical administration of ACh causes response facilitation or suppression in individual V1 neurons. The proportion of the type of response modulation (facilitation, 35% (53/153 cells); suppression, 32% (49/153 cells)) was similar to our recent study using ionophoretic administration of ACh in rat V1 where facilitation and suppression were observed in 40% (39/99 cells) and 28% (28/99 cells) of all recorded cells, respectively [26]. However, the laminar distribution of the ACh effect was obviously different between the two studies. Ionophoretic administration caused no laminar bias of the ACh effects, whereas topical administration did, with the response suppression and facilitation prevailing in *layers 2/3* and *layer 5*, respectively (see Fig. 3). This finding appears independent of the ACh concentration gradient, because sufficient levels of topically administered ACh reached the deep layers of V1 (see Fig. 4), and because the concentration did not affect the direction of the ACh effects (see Fig. 5).

What is the reason for the discrepancy between our present and previous studies [26]? Since the experimental protocols for maintaining animals including anesthesia were identical, the most probable reason was the difference in the diffusion area of ACh. Ionophoretically administered drug diffuses about several hundred micrometers, affecting only a limited local circuit [42]. On the other hand, topically administered ACh prevails throughout all cortical layers and affects several millimeters within V1 in which intra- and inter-laminar signal processing and transmission are modulated. Indeed, Goard and Dan [27] observed a similar laminar bias of ACh effects by BF stimulation in rat. They examined the effects of intrinsically released ACh on visual responses by directly stimulating the BF, finding like us that a suppressive effect was mainly observed in *layers 2/3* and a facilitatory effect was dominant in *layer 5* (Supplementary Fig. 2 of Goard and Dan [27]). Since BF stimulation causes ACh release into various cortical areas including V1 and higher visual areas, it is possible that the ACh effects observed in V1 inherited feedback inputs from areas other than V1. Our data suggest that the laminar bias of ACh effects can be evoked by at least the direct action of ACh on intra- and inter-laminar networks within V1. Therefore, we will discuss the present results from the viewpoints of the direct ACh effects on networks of V1 especially focusing on suppressive modulation in *layers 2/3* and facilitatory modulation in *layer 5* in what follows.

The prevalence of suppressive modulation in *layers 2/3* might be explained by activation of a specific type of inhibitory interneuron. Using two-photon imaging and genetically manipulated mice to visually identify different subtypes of inhibitory interneurons, Alitto and Dan [43] found in V1 *in vivo* that when BF is strongly activated, widely released ACh activates vasoactive intestinal peptide-positive (VIP^+^) neurons in *layers 2/3* and interneurons in *layer 1*, and thereby both excitatory neurons and parvalbumin-positive (PV^+^) neurons are strongly suppressed. On the other hand, when BF activation was weak, the VIP^+^ neurons were only weakly activated, and the excitatory neurons were activated through AChRs. This explains well why the suppression was dominant when *layers 2/3* were widely affected by topically administered ACh, but not locally affected by ionophoretically administered ACh [26]. A recent study in cortical slices of mice V1 also demonstrated that optogenetic stimulation of cholinergic fibers originating from the BF causes nicotinic AChR (nAChR)-mediated excitation of interneurons in *layer 1* and non-FS interneurons in *layers 2/3* [44]. Moreover, Gullege et al. [45] reported that ACh depolarizes non-FS (VIP^+^ or cholecystokinin-positive) interneurons, but not PV^+^ interneurons. Thus, a subtype of inhibitory interneurons other than PV^+^ interneurons seems to play an important role in generating the prevalence of suppressive modulation in *layers 2/3*. Another possibility explaining this prevalence is the direct suppression of excitatory neurons via AChRs. In fact, *layer 3* excitatory neurons expressing muscarinic AChRs (mAChRs) are inhibited by ACh via direct activation of a potassium conductance [45]. More experiments are needed to clarify how the suppressive modulation arises.

Combined with our previous report [26], our results demonstrate that neurons in *layer 5* are predominantly facilitated by topically administered ACh in this study, but not if the ACh is ionophoretically administered. The differential diffusing area of ACh may explain the different effects. Kena-Vaknin et al. [46] found that microdrop administration of ACh onto *layers 2/3* or *6* cells causes excitatory postsynaptic potentials (EPSPs) in *layer 5* pyramidal cells. The generation of EPSPs was abolished by tetrodotoxin, suggesting that the excitatory action of ACh is indirect. On the other hand, ACh administered near pyramidal cells in *layer 5* caused a transient hyperpolarization of these cells that was associated with a decrease in input resistance. These results suggest that ACh activates *layer 5* pyramidal cells indirectly via activation of *layers 2/3* or *layer 6* cells and inhibits directly. Therefore, topically administered ACh is thought to facilitate *layer 5* neurons indirectly via activation of neurons in other layers, whereas ionophoretically administered ACh seems to inhibit them directly.

ACh has been reported to facilitate the release of transmitters from both excitatory and inhibitory synapses to *layer 5* cells by activating presynaptic sites via AChRs [47]. Therefore, another explanation for the differential laminar distribution of ACh effects is a differential localization of excitatory and inhibitory synapses. *Layer 5* pyramidal cells possess long apical dendrites spanning *layers 1–5*, and excitatory and inhibitory synapses are known to locate differentially on the dendritic trees [48]. More specifically, inhibitory synapses are frequently found on the dendritic trunks or on the somata, whereas excitatory synapses are mainly formed on the head of the dendritic spines or at proximal and distal dendritic shafts. Therefore, local ionophoretic administration of ACh around the soma is likely to promote inhibitory transmission, while topical administration of ACh affects both excitatory and inhibitory transmission, causing a balance of their inputs to shift toward excitation-dominant. Further study is required to clarify these possibilities.

### Facilitatory ACh effects on gain control

ACh has been known to exert its facilitatory modulation via two distinct AChR subclasses, nAChRs and mAChRs [49]. Recent neuropharmacological studies using primates have directly shown that both AChRs are responsible for response gain control [17], [18], [20]. Interestingly, contribution of the receptor subclasses showed a laminar bias in which nAChRs mainly operated in the thalamocortical layer, and mAChRs operated across all cortical layers. These physiological observations are in accordance with the laminar distribution of corresponding receptor subclass proteins in primate V1, where nAChRs are predominantly expressed in *layer 4*, and mAChRs are observed without laminar bias [17], [50]. The same laminar bias of receptor subclass proteins has been reported in rodent V1 [51], [52], but no corresponding neuropharmacological study using antagonists specific for nAChRs or mAChRs has been reported. Thus, further study is needed to confirm how each receptor subclass contributes to ACh effects in individual cortical layers.

### Suppressive ACh effects on spatial phase sensitivity

In suppressed neurons in *layers 2/3*, the F1/F0 ratio was increased by ACh, meaning that neurons became more sensitive to the spatial phase of a visual stimulus. This result can be explained well by the network model with cortical amplification proposed by Chance et al. [53]. There, complex cell responses arise as a consequence of decreasing the phase selectivity of simple cell responses by recurrent intracortical connections. Neurons exhibit simple-cell-like responses when recurrent connections are weak and complex-cell-like responses when they are strong. Therefore, the model predicts that a decrease in intracortical excitation should cause complex cells to respond like simple cells [53]. Experimental evidence supports such a conclusion [54]. Indeed, ACh strongly suppresses intracortical connectivity through presynaptic mAChRs [55]. Consistent with these reports, ACh in the visual cortex sharpens visual receptive field tuning by reducing spatial integration [19]. Therefore, the improved F1/F0 ratio of *layers 2/3* neurons seems to be the result of suppressed intracortical connectivity.

### Functional role of cholinergic modulation

The relationship between the response modulation type and the laminar position of a cell provides important information about the functional role of ACh in visual information processing, since the modulated output from each layer goes to its own destination layers and areas. The response suppression was predominantly observed in *layers 2/3*, whose neurons project to higher-order visual areas. This hierarchical feedforward information processing is essential for visual recognition. In suppressed cells, ACh reduces the response gain by decreasing more preferentially the F0 component of the visual response than the F1 component, resulting in an increase in the F1/F0 ratio. This means that grating-phase-independent responses are attenuated more strongly by ACh than grating-phase-dependent responses. Moreover, these ACh effects were observed in RS cells (presumed output cells), but not in FS cells (presumed inhibitory interneurons). Thus, ACh suppressive effects do not simply reduce the output from V1. Rather, they promote the transmission of a particular signal, grating-phase information, to higher-order visual areas.

Meanwhile, the fraction of response facilitation was high in *layer 5*, whose neurons project fast-conducting descending axons to subcortical visual areas including the superior colliculus (SC). Corticotectal neurons in *layer 5* acts as potent drivers of the SC [56], and the corticotectal pathway plays an important role in the generation of visually guided saccadic eye movements [57]. In fact, focal and electrical microstimulation of *layer 5* evokes a saccade toward the retinotopically corresponding visual field [58]. Interestingly, facilitated RS and FS cells showed different modulatory changes in their S/N ratio, as the S/N ratio was improved in RS cells, but not FS cells. Therefore, ACh released into V1 seems to facilitate visually guided saccadic eye movements by enhancing excitatory inputs with a high S/N ratio from *layer 5* to the SC.

To conclude, ACh has distinct effects on functionally differentiated cortical layers, suggesting laminar-dependent modulation. It is released context-dependently, especially when animals explore their environment to obtain detailed information [3]. Therefore, ACh in V1 would appear to improve visual performance in two ways. One, by directing the eyes toward an attracting object by facilitating *layer 5* neurons; and two, by transmitting the phase information of an object by suppressing *layers 2/3* neurons.

### Advantages and disadvantage of different methods for ACh and drug delivery

Although there are several methods for examining the functional roles of ACh in visual information processing *in vivo*, each has its advantages and disadvantages. For example, the electrical or optogenetic activation of cholinergic neurons in the basal forebrain is suitable for mimicking the natural pathway of ACh release, but the action mechanisms responsible for the observed effects cannot be easily elucidated. Since ACh affects a variety of cortical areas, it is difficult to discriminate direct action on V1 neurons from indirect action via neurons in other visual areas. In the case of micro-electrical stimulation, electrical effects are not limited to target nuclei or target neurons.

Microionophoretic administration is suitable for examining the direct action of ACh on target neurons or target areas. However, since ACh affects only a local circuit, it is not clear how visual responses are affected by more widely released ACh like that which occurs in natural conditions. Topical administration may be better for assessing the modulatory effects of ACh in these cases [1], [27]. The influence of ACh on a non-target cortical area can be minimized by controlling the administration condition. Nevertheless, unexpected effects due to excess ACh, like the desensitization of ACh receptors, should be taken into account. However, since the direction of response modulation was independent of the intensity of the ejecting current (concentration of ACh; see Fig. 5), the type of response modulation seen here is unlikely to be explained by receptor desensitization.
